# Repositioned Natural Compounds and Nanoformulations: A Promising Combination to Counteract Cell Damage and Inflammation in Respiratory Viral Infections

**DOI:** 10.3390/molecules28104045

**Published:** 2023-05-12

**Authors:** Alessia Mariano, Irene Bigioni, Magda Marchetti, Anna Scotto d’Abusco, Fabiana Superti

**Affiliations:** 1 Department of Biochemical Sciences, Sapienza University of Rome, 00185 Rome, Italy; 2 National Centre for Innovative Technologies in Public Health, National Institute of Health, Viale Regina Elena 299, 00161 Rome, Italy

**Keywords:** natural compounds, respiratory viral infections, nanotechnology, inflammation, cell damage

## Abstract

Respiratory viral diseases are among the most important causes of disability, morbidity, and death worldwide. Due to the limited efficacy or side effects of many current therapies and the increase in antiviral-resistant viral strains, the need to find new compounds to counteract these infections is growing. Since the development of new drugs is a time-consuming and expensive process, numerous studies have focused on the reuse of commercially available compounds, such as natural molecules with therapeutic properties. This phenomenon is generally called drug repurposing or repositioning and represents a valid emerging strategy in the drug discovery field. Unfortunately, the use of natural compounds in therapy has some limitations, due to their poor kinetic performance and consequently reduced therapeutic effect. The advent of nanotechnology in biomedicine has allowed this limitation to be overcome, showing that natural compounds in nanoform may represent a promising strategy against respiratory viral infections. In this narrative review, the beneficial effects of some promising natural molecules, curcumin, resveratrol, quercetin, and vitamin C, which have been already studied both in native form and in nanoform, against respiratory viral infections are presented and discussed. The review focuses on the ability of these natural compounds, analyzed in in vitro and in vivo studies, to counteract inflammation and cellular damage induced by viral infection and provide scientific evidence of the benefits of nanoformulations in increasing the therapeutic potential of these molecules.

## 1. Introduction

Respiratory viruses are among the most frequent agents responsible for illness in humans, occurring in all age groups worldwide [1,2]. The severity of these diseases can vary markedly from mild or asymptomatic self-limiting infections, confined to the upper airways, where they evoke relatively minor symptoms such as sneezing, to very severe manifestations with wheezing, bronchiolitis or pneumonia [3]. In particular, severe symptoms have often been associated with overwhelming inflammation, mediated by a cytokine storm, which is responsible for the acute respiratory distress syndrome (ARDS) and lung failure [4,5,6]. As also seen during the COVID-19 pandemic, fragile patients, children and the elderly have a higher risk of developing these complications, leading to hospitalization or death [7,8,9].

During the last few years, the severe acute respiratory syndrome generated by Coronavirus-2 (SARS-CoV-2) infection has captured global attention, highlighting the strong impact of respiratory viruses on global health [10,11]. During this pandemic, among other criticisms, it emerged that the therapeutic options to fight viral respiratory infections are very scarce. Therefore, the development of novel and more efficacious therapeutic strategies is the greatest challenge for global health.

In addition to the development of novel therapies targeting viral infection, in recent years, the drug-repositioning phenomenon, also known as drug repurposing, has been widely considered a viable low-cost and time-saving alternative. It consists of finding effective molecules for viral disease treatment using existing drugs approved for other clinical indications [12]. Facing such challenging research, natural molecules have been proven to be an excellent alternative therapeutic approach and an interesting source for antiviral drug development or repositioning [13]. Compared to marketed drugs, natural molecules are often easier to obtain and have fewer side effects while still showing good pharmacological properties and applications in different branches of medicine, from inflammatory to infectious diseases [14,15,16,17,18]. On the other hand, the low bioavailability of these compounds is their major limiting factor. They generally exhibit poor kinetic performance due to characteristics such as their large molecular weight, inability to cross lipid membranes, and weak absorption capacity, which finally results in reduced therapeutic effect [15].

In this respect, the advent of nanotechnology in medicine in recent years represents an excellent tool with which to improve the effectiveness of natural compounds in the treatment of several diseases as it increases their bioavailability [19]. Specifically, nano-based drug delivery systems provide a large surface area that increases dissolving properties, while also facilitating lipid membrane crossing in a way that is dependent on the nanosystem’s features [20,21]. For this reason, the combination of nanotechnology with natural products has very broad therapeutic application prospects, becoming increasingly popular in the pharmaceutical field.

In this narrative review, we analyzed the anti-inflammatory effect of some “repositioned” natural compounds against respiratory viral infections, focusing on the advantages of nanoformulations in in vitro and in vivo studies. Although there are multiple natural substances with anti-inflammatory properties against viral respiratory infections, in this narrative review, only curcumin, resveratrol, quercetin, and vitamin C were examined. The reason for this choice is represented by their wide use in food supplements and nutraceuticals administered as immunomodulators and antioxidant/anti-inflammatory compounds. To date, only few studies have been conducted on molecules in nanoform. However, research on curcumin, resveratrol, quercetin, and vitamin C nanoformulations has been performed, allowing a comparison between them and their native form. These natural compounds in nanoform can provide a new platform for the manufacture of bio-safe and efficient treatments for respiratory diseases of viral origin.

## 2. Inflammatory Response to Respiratory Viral Infections

Through analysis of the pathogenesis of viral respiratory infections that have attracted global attention in recent years, such as those due to SARS-CoV, MERS-CoV, the pandemic Influenza A (H1N1) 2009 virus, avian Influenza A (H5N1) virus and SARS-CoV-2, the recurrent presence of virus-induced hyperinflammation has been revealed [22]. In particular, several scientific studies have shown a correlation between this virus-induced hyperinflammation and the development of severe symptoms [6,23,24]. Specifically, they have demonstrated that this hyperinflammatory response shares many biological and clinical components with immune-mediated diseases, suggesting a significant involvement of the immune system of patients following respiratory viral infections [25].

Respiratory viruses primarily target and infect the upper and/or lower respiratory tract, so during their replication in human cells, they cause the release of damage-associated molecular patterns (DAMPs) and pathogen-associated molecular patterns (PAMPs), from host cells and viruses themselves, respectively [26,27,28]. These patterns are recognized by pattern recognition receptors (PRRs) present on lung epithelial cells, endothelial cells, and alveolar macrophages, triggering the generation of pro-inflammatory cytokines and chemokines [29]. PRRs include families of cell-membrane-bound and cytoplasmic receptors, which provide a first-defense line against pathogens. Once DAMP-PRR signaling is activated, cells produce an array of pro-inflammatory cytokines, such as IL-6, IL-1β, TNF-α, and interferons, attracting neutrophils and monocytes as well as other inflammatory cells into the respiratory tract [30].

This cytokine overproduction, defined as a cytokine storm, and the increase in inflammatory infiltrates causes lung edema, reducing alveolar gas exchange, and leading to ARDS, which is responsible for lung failure [31,32]. Analyses of the serum of patients with acute lung damage and multi-organ failure, infected with respiratory viruses, including the pandemic H1N1 2009 and A/H5N1 Influenza viruses, coronaviruses such as SARS-CoV-2, SARS-CoV and MERS-CoV, respiratory syncytial virus (RSV), and rhinovirus, revealed an increase in pro-inflammatory cytokines (e.g., IL-1, IL-6, TNF-α and interferons) that are considered mainly responsible for an unfavorable prognosis (Table 1) [22,33,34]. Therefore, considering this role of the immune response, in addition to antiviral therapies that can directly affect the virus, anti-inflammatory therapies that target the cytokine storm can be considered a novel and promising treatment strategy with which to relieve this excess influx of cytokines into the infection site to avoid serious consequences [35].

### The Interplay between Oxidative Stress and Inflammation in Viral Infections

Respiratory viruses induce redox stress, altering the balance between increased production of reactive oxygen species (ROS) and reduced antioxidant host responses [48]. Increased ROS production during infection alters immune functions and induces inflammatory responses, lung epithelial disruption and organ dysfunction [49]. Many studies demonstrated the contribution of oxidative stress to the pathogenesis of respiratory viral infections [50]. For example, increased ROS production in severe COVID-19 patients triggers inflammation, endothelial cell dysfunction, and thrombosis leading to multiorgan failure [51]. Moreover, coronavirus-induced oxidative stress, interfering with inflammatory pathways, can cause long-lasting tissue damage [52].

Concerning inflammation, it is well-known that respiratory viruses induce NF-κB, a key mediator of cytokine induction, in a ROS-dependent manner [53]. Indeed, both NF-κB activation and cytokine production induced via rhinovirus infection are reduced with low-molecular-weight antioxidants, demonstrating that ROS are mediators of both effects. Inflammation and cell injury are strictly related as cell lung damage can promote inflammation and vice versa [50]. Regarding the role of ROS in cell damage, high ROS production in lung epithelial cells can result in a loss of mitochondrial function and apoptosis [54]. In particular, in COVID-19, these effects are associated with lung injury [55]. ROS-induced cell lysis promotes viral release favoring the spread of cytolytic respiratory viruses.

In summary, in respiratory infections, ROS play a major role in inflammation, cell damage and viral spread, and antioxidant therapies are able to improve disease outcome [50].

## 3. Treatment of Respiratory Viral Infections

The severity of the viral respiratory disease determines the therapeutic approach. The treatment of low-risk respiratory viral infection is often prevalently supportive. In other words, healthy adults and children preferentially resort to the use of nonsteroidal anti-inflammatory drugs such as aspirin, naproxen, diclofenac sodium, and ibuprofen to reduce symptoms, including fever, headache, and aches in the muscles. Instead, for patients with severe or progressive illness, who are at a high-risk of virus-associated complications, or who are hospitalized, prescription of antivirals is recommended. Patients should start antiviral treatment as soon as possible, and the greatest clinical benefit occurs within 24 h of symptom onset, considerably reducing the risk of complications [56].

To date, few antiviral drugs are currently approved for the treatment of respiratory virus infections, most of which are specific against Influenza A viruses and RSV [57]. For this reason, also considering the global health emergency of the last few years, numerous studies are aimed at developing new therapies for other respiratory viruses. In particular, several pre-clinical and clinical studies have been approved, and are still ongoing, to develop drugs against new viral targets, to increase potency and reduce resistance, also mitigating the strong responses of the host immune system [58,59,60,61]. Nevertheless, the processes of new drug discovery are very difficult. They require long times, high costs, and numerous steps (from target identification to post marketing surveillance) before arriving at an effective molecule [62]. For this reason, the drug-repurposing or drug-repositioning approach is considered an emerging strategy with which to identify existing drugs for new therapeutic indications within a short period of time [63]. Considering the high level of global health emergency, this approach was widely used during the COVID-19 pandemic given the need for valid therapeutic treatment. Indeed, the first medical approaches were based on clinical analysis of the activity of antivirals such as Remdesivir, Favipiravir and antimalarial drugs such as Chloroquine and Hydroxychloroquine, to control pathological events caused by COVID-19 [64,65,66,67]. This approach was also widely used to control the immune-mediated hyperinflammatory response, typical of several viral infections, including SARS-CoV-2 infection. A number of immunomodulatory and anti-inflammatory agents, such as Tocilizumab (a monoclonal antibody against IL-6 receptors normally used for the treatment of rheumatoid arthritis), Baricitinib, Ruxolitinib, Eculizumab (that are Janus kinase (JAK) inhibitors), and corticosteroids, have been “repositioned” and tested to counteract the cytokine storm, in parallel with antiviral drugs [68].

Apart from marketed drugs, many natural molecules have also been studied to treat or manage symptoms of respiratory viral infections. In recent years, studies describing the positive effects of natural products, mainly extracted from plants, in the treatment and healing of various pathologies have been numerous [69,70,71,72], and also in the field of virology, several phytochemicals and phytocomplexes have been screened to test their effectiveness [73,74,75]. Moreover, compounds of natural origin are very well-placed in the drug repositioning strategy, as they are characterized by unique properties due to their various phytochemical composition to which many pharmacological activities are attributed. Therefore, they play a key role in drug discovery strategies, as they can have new therapeutic utilities unrelated to their original biological use with remarkable advantages over conventional molecules [76,77]. In this regard, flavonoids, polyphenols, alkaloids and several other natural compounds, traditionally used to treat infectious diseases for their immuno-suppressing, antibiotic and anti-inflammatory properties, have been repurposed in clinical studies for the treatment of COVID-19 to counteract the cytokine storm and to modulate the immune inflammatory response [78,79,80,81].

**Figure 1:**
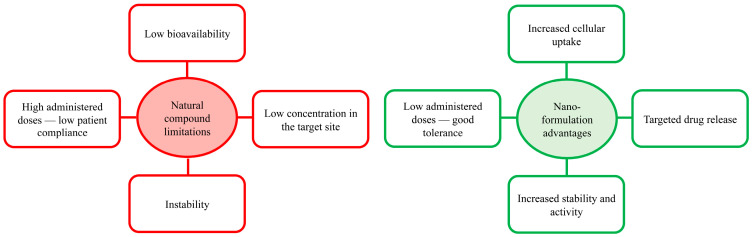
Schematic representation of the limitations of natural compounds and nanoformulation advantages.

Compared to conventional drugs, natural compounds have several advantages, including different therapeutic indications for the presence of numerous bioactive substances, a low cost, and a higher safety profile. On the other hand, their pharmacokinetic profile is considered the biggest limit of natural drug applications in the treatment of various diseases. Some of the most common bioactive compounds, such as flavonoids, polyphenols, tannins and terpenoids, indeed, show higher solubility in water and have a higher molecular size [82,83,84]. This makes it difficult for them to be absorbed through cell membranes, generating poor metabolism and rapid excretion, ultimately resulting in reduced therapeutic activity. Accordingly, to achieve the desired beneficial effect, it is necessary to administer a higher dose of medication, often leading to a decrease in patient compliance [85,86]. In this context, nanotechnology represents a new therapeutic approach to overcome these limitations (Figure 1).

## 4. Nanotechnology

Nanotechnology usually refers to the use of nanoparticles (NPs) and more generally materials of up to 500 nm with at least one size in the range of 1 to 100 nm, which can be classified according to different preparation techniques [87,88,89]. The most commonly used strategies include drug placement inside organic or inorganic materials, physically enveloping these molecules, forming NPs widely used in drug delivery. Types of nanocarrier mainly include organic and polymeric NPs, nanoemulsions, solid lipidic NPs, and inorganic or metallic NPs [90,91]. Each of these systems has unique properties and therefore can be selectively designed for specific medical applications.

Organic NPs are among the most common systems in drug delivery, accounting for more than two thirds of all the available nanosystems. The presence of organic molecules, indeed, allows the greater chemical variability in surface composition with various combinations, dimensions, forms, and types of functionalization of the systems, which make them useful in different fields of medicine [92]. Moreover, although these are less stable than inorganic NPs, particularly at high temperatures or pressures, they still exhibit excellent biocompatibility, stability, and efficacy in drug transport and release [93].

Inorganic NPs are composed of a wide range of substances including elemental metals, metal oxides (e.g., silver, gold, iron oxide, zinc oxide and silica) and carbon-based NPs, including fullerenes, graphene, and carbon nanotubes [94]. They have unique characteristics and relevant physical properties that make them suitable for application in medicine and more specifically for imaging techniques and for drug administration [95]. However, despite these advantages, only a limited number of inorganic NPs are translated into clinical practice. This is mainly due to the lack of sufficient evidence and data regarding their biosafety, especially their biodegradation behavior, excretion routes and long-term toxicity assessments [96,97]. Among the inorganic NPs, in particular metal-containing or metal-oxide NPs, have been shown to cause oxidative stress in the liver, spleen and kidneys, generating reactive oxygen species (ROS) and inducing DNA damage and protein structure modification [98,99,100,101]. Thus, the discovery of strategies with which to limit the “nanotoxicity” of inorganic NPs has been the subject of numerous investigations of recent years. Several scientists have turned their research towards alternative methods of synthesis and design of nanosystems with a safe and biocompatible approach using green materials or natural molecules as an alternative to less healthy synthetic or metal-containing products [102,103,104].

**Figure 2:**
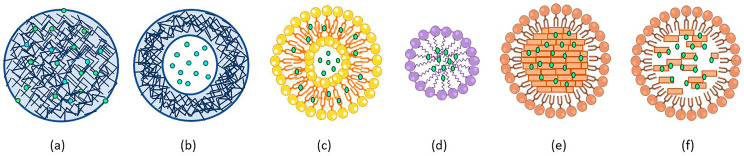
Schematic illustration of organic NP systems. Polymeric NPs such as nanospheres (**a**) and nanocapsules (**b**), liposomes (**c**), micelles (**d**), solid lipid NPs (**e**) and nanostructured liquid carriers (**f**) are represented by loaded drugs (green dots).

For all these reasons, organic NPs are generally more commonly employed. In this part of the narrative review, some of the most used organic NPs, used as delivery systems for drugs against respiratory viral infections, such as polymeric NPs, liposomes, micelles, and solid lipid NPs, will be described (Figure 2; Table 2).

### 4.1. Polymeric Nanoparticles

Polymer-based NPs are colloidal systems made up of natural or synthetic polymers in which the therapeutic agent is dissolved, entrapped, encapsulated, or adsorbed onto the surface [109,110]. Depending on the production process, the active substance is differently arranged and both nanocapsules and nanospheres can be obtained. In nanocapsules, the drug is usually dissolved inside an oily core surrounded by a thin polymeric matrix envelope that regulates its release, and therefore they are considered reservoir systems. While nanospheres are based on a continuous solid polymeric network where the drug can be kept or adsorbed onto the surface [111]. In drug delivery systems, encapsulation of drugs in polymeric NPs has several advantages. On one hand, drugs are protected from destruction, degradation, and rapid metabolism; on the other hand, the organism is preserved from the release of drug toxic metabolites. Moreover, both the encapsulation of drugs in nanocapsules and their loading on nanospheres allows to target the drug release improving its delivery and bioavailability in a specific site of action. These polymeric nanostructures, indeed, are slowly degraded through enzymatic action, allowing controlled drug release [112].

The first polymeric NPs used in therapy were made using synthetic polymers such as polyacrylamide, polystyrene or polymethylmethacrylate, which are easy to obtain and manufactured at a large scale. However, chronic or prolonged administration of these NPs has been linked to systemic toxicity, and allergic and immunological responses, requiring a reduction in their use. Because of these reasons, natural and biodegradable polymeric NPs have become more important over the years. The most commonly used natural polymers in the preparation of polymeric NPs are chitosan, gelatin, sodium alginate, hyaluronic acid or albumin [113,114]. In addition to the less-systemic responses, once degraded by enzymatic processes, they do not require clearance from the body, making them an appropriate choice for drug delivery systems [115,116,117].

### 4.2. Liposomes

From point of view of terminology, the word liposome is derived from two Greek words: *lipos* (λίπος) and *soma* (σώμα), which are fat and body, respectively, and indicate its structural composition [118]. Liposomes, indeed, are self-assembled phospholipid vesicles consisting of one or more concentric lipid bilayers (lamellas) that enclose a central aqueous compartment. Due to this peculiar structure, they are able to encapsulate both lipophilic and hydrophilic compounds, allowing the transport of a wide range of drugs. In particular, lipophilic molecules are inserted into the bilayer membrane, while hydrophilic molecules are entrapped in the aqueous core [119,120]. This amphiphilic feature is one of the major advantages of the use of liposomes in drug delivery systems. Moreover, liposomes are usually composed of cholesterol and phospholipids such as phosphatidylcholine, phosphatidylserine, and dimethyl phosphate, with a composition, proportions, and characteristics similar to those of cell membranes [121]. This structure allows effective interactions among the system and cell membranes increasing absorption and targeted drug release.

### 4.3. Micelles

Micelles are amphiphilic colloidal nanosized structures with a hydrophobic core and hydrophilic shell. They are obtained via the autonomous aggregation of amphiphilic molecules, such as polymers, at a specific temperature, known as critical micellar temperature, and at a certain concentration, known as critical micellar concentration (CMC), which depend on the size and composition of the system [122]. Their structure allows good solubilization of hydrophobic drugs into the hydrophobic core, isolating them from the surrounding environment with the polymer shield, and protecting them from a rapid metabolism, increasing their blood half-life [123]. Compared to other nanocarriers, polymer micelles generally have a smaller size that allows more efficient cellular internalization, including through amphiphilic polymer interactions with biological membranes. In addition, comparing them with polymeric NPs and liposomes that need long expensive and complex production procedures, they require simple preparation processes, improved sterilization stability and good industrial reproducibility, which make them great alternatives in the drug delivery field [124].

### 4.4. Solid Lipid Nanoparticles and Nanostructured Liquid Carriers

Solid lipid NPs belong to the second generation of NPs and have been designed to replace liposomes, micelles and polymeric systems [125]. These are composed of a lipid core matrix, which is solid at room temperature as well as at body temperature, that is stabilized by surfactants or emulsifiers. The lipid core is mainly composed of triglycerides, fatty acids (stearic acid and palmitic acid), steroids (cholesterol) or waxes (cetyl palmitate), while different emulsifiers are used to stabilize the lipid phase.

The use of solid lipids instead of liquid oils represents one of the major advantages of employing this type of system. These have good stability and controlled drug release at the target site due to the low drug mobility in a solid lipid matrix [126]. On the other hand, solid lipids represent a limit in drug incorporation: they minimize water accumulation into the system and lead to the formation of lipid crystals which recognize each other over time. This is a typical feature of lipids which tend to rearrange in the space to achieve a more organized structure with less energy. This lipid reorganization presents less available space for drug loading, since molecules tend to fit into the imperfections of the lipid matrix or between the fatty acid chains [127]. For these reasons, in recent years, another class of lipid NPs, called nanostructured liquid carriers, prepared using a mixture of solid lipids and liquid oils, has been developed. The presence of liquid oils reduces the crystal rearrangement, minimizing drug expulsion during storage and increasing the ability to encapsulate them. For their composition, these lipid NPs have a lower tendency to achieve an organized disposition, retaining imperfections in the lipid matrix where molecules are located [125,128].

## 5. Natural Products and Nanotechnological Approach to Treating Respiratory Viral Infections

**Figure 3:**
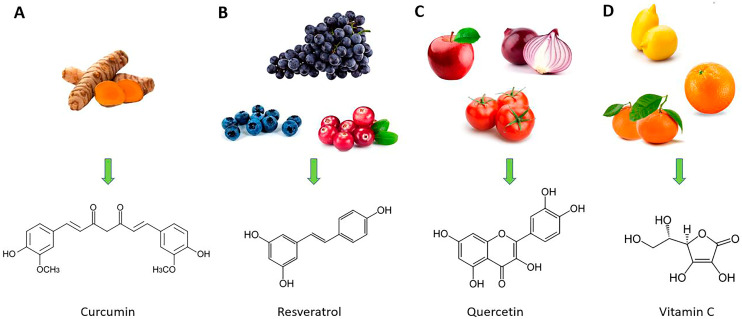
Natural sources of curcumin (**A**), resveratrol (**B**), quercetin (**C**), and vitamin C (**D**) (at the top of the figure) and their chemical structures (at the bottom of the figure).

In recent years, the study of the therapeutic benefits of natural bioactive substance-based nanomedicine in the treatment of various diseases, including respiratory viral infections, has attracted more and more attention [129,130,131,132,133,134,135]. These pathologies are characterized by acute or chronic inflammatory processes and high oxidative stress in which medicine based on nanotechnology and natural compound association seems to have a promising future [91]. For these reasons, the purpose of this narrative review is to describe the properties of some of the most commonly studied “repositioned” natural compounds, such as curcumin, resveratrol, quercetin, and vitamin C (Figure 3), in the treatment of inflammation caused by respiratory virus infections, highlighting the advantages of their use as nanoformulations.

### 5.1. Curcumin

*Curcuma longa* is a member of the *Zingiberaceae* (ginger) family whose rhizome has been used for centuries for medical applications, particularly those against inflammatory diseases [136]. Additionally, it was employed to treat several other illnesses such as neurological disorders, asthma, and diabetes, and as an antiseptic. *C. longa* contains various constituents including terpenes, phenolic compounds, steroids, fatty acids, and other molecules. Among them, curcumin is the major constituent that is well-known for its therapeutic potential in all of these disorders [137]. Several studies conducted with cell cultures and animal models have demonstrated that curcumin is able to modulate the activity of many molecular targets, including transcription factors, enzymes, and growth factors, and to interfere with a multitude of signaling pathways involved in inflammation [138,139].

For these reasons, taking into account its wide spectrum of action, curcumin has been largely considered a promising candidate in the prophylaxis and treatment of viral infections. In recent years, several studies have tested the effect of curcumin against respiratory diseases from Influenza A virus (IAV), RSV and other respiratory enveloped viruses including SARS-CoV, also demonstrating, in addition to an antiviral effect, the ability to modulate the immune response and the cytokine storm [140,141,142,143,144]. The evidence comes from studies carried out on animal models in which it was observed that treatment with curcumin can reduce the symptoms associated with these respiratory viral infections, reducing acute respiratory distress syndrome (ARDS), pulmonary lesions and fibrosis by modulating inflammation and oxidative stress, finally improving prognosis [145,146,147]. Given the benefits, curcumin has been recognized by the USA Food and Drug Administration (FDA), as a product with good tolerance and safe administration (generally recognized as safe—GRAS) [148], and has been used in some clinical trials in COVID-19 patients with moderate and severe forms of infection [149]. These studies demonstrated that curcumin treatment reduces the hyper-inflammatory effect in patients by restoring the balance between pro-inflammatory and anti-inflammatory cytokines and, consequently, mitigating the persistence of symptoms [150]. However, the highest limit in these clinical trials is given by the pharmacokinetic characteristics of curcumin which confine its use in therapy. Indeed, curcumin shows poor bioavailability with low or undetectable concentrations in the blood for poor intestinal absorption, fast metabolism and rapid systemic clearance, both in human and in animal models [151,152,153,154].

In order to overcome these pharmacokinetic limitations, a number of curcumin nanoformulations have been developed. In a recent in vitro study, Sharma et al. evaluated the anti-inflammatory efficacy of encapsulated curcumin in polymeric NPs, demonstrating the advantages of this formulation over the bulk form, in a virus-induced cytokine storm [155]. They have highlighted that the treatment of SARS-CoV-2-infected human alveolar basal epithelial cells A549 with curcumin NPs is more effective at reducing the release of IL-6 and IL-8 pro-inflammatory cytokines, which are responsible for lung damage, and at inhibiting the activation of MAPK and NF-κB signal pathways by interfering with its factor phosphorylation. These findings have been confirmed in a human clinical trial [156]. The use of polymeric NPs was also considered in the in vitro study conducted by Gunathilake and colleagues [157]. These researchers began with the idea of Zachar et al. to develop NPs administered via inhalation in order to increase the bioavailability of the curcumin at the lung level [158]. Compared to the Zachar group that developed silver-based inorganic systems, Gunathilake et al. proposed cellulose NPs for intranasal curcumin delivery [157]. Nanocellulose, indeed, has the advantage of improving loading capacity and permeability through mucus barriers in the airway, enhancing cellular uptake, and increasing lung retention, also reducing the chronic health effects of silver NPs. Moreover, the development of inhalation formulations is also the strategy chosen by another research group to increase the therapeutic effect of anti-inflammatory molecules in ARDS [159]. In this study, curcumin-containing functionalized liposomes were designed to target M1 macrophages at the lung level. Macrophages are highly involved in the immune response with a key role in the cytokine storm, representing excellent targets for therapy. Functionalized liposomes showed numerous advantages both in vitro and in vivo; they increased curcumin uptake by M1 macrophages in vitro in RAW 264.7 cells and allowed higher macrophage accumulation in acute lung injury rat models, enhancing the curcumin anti-inflammatory effect [159].

The pharmacokinetic advantage of the use of nanoformulations was also highlighted in other in vitro studies, including one by Li et al. [160]. In this research, curcumin-loaded self-assembled micelles demonstrated considerable benefits over the molecule in bulk form. The encapsulation of the molecule significantly increased its bioactivity and stability, allowing it to counteract IAV infection and its replication in host cells in a more potent way than achievable with native curcumin. In addition, the authors also highlighted its simplicity and low cost in self-assembled micelle preparations, proposing these formulations as an excellent resource for oral drug administration against viral activity.

### 5.2. Resveratrol

Resveratrol is one of the most well-studied natural polyphenols and mainly abounds in the skin and seeds of grapes, but can be also detected in more than 70 plant species [161]. In plants, it acts as a phytoalexin that is synthesized in response to mechanical lesions, UV irradiation and fungal infections, as a means of protection for the plant itself [162]. However, over the years, numerous scientific publications have highlighted the protective properties of resveratrol even in human health. Different pharmacological activities have been attributed to this polyphenol, including antioxidant, anti-aging, anti-inflammatory, anti-cancerous, cardioprotective and neuroprotective activities, which have increased industry interest in the nutraceutical field [163].

Palamara and co-workers described for the first time in 2005 a specific antiviral activity of resveratrol in addition to its antioxidant and anti-inflammatory potential in IAV infection [164]. They examined this molecule for its known ability to regulate the cellular redox state, which is highly altered in viral infections and concomitant lung inflammation. Resveratrol was initially thought to be able to restore glutathione antioxidant potential and, as a result, to modulate the viral proliferation. Surprisingly, they demonstrated that resveratrol is also responsible for the inhibition of nuclear–cytoplasmic translocation of viral ribonucleoproteins in MDCK cells, reducing late viral protein expression, and inhibiting PKC activity and its dependent pathways involved in virus replication. For this reason and for its enormous health benefits, resveratrol has been the subject of several studies in recent years as an adjuvant treatment for strengthening the immune system during viral infection and fighting the cytokine storm. This research has shown that resveratrol interferes with several signaling pathways in inflammation. In vitro and in vivo studies on ARDS models have shown that the polyphenol interferes with the activation of the NF-κB-mediated inflammatory pathway, with the MAPK signaling cascade downstream reducing TNF-α, IL-6, and IL-1β secretion in lungs [165,166]. Moreover, in pneumonia and RSV models, resveratrol was able to modulate the expression of Toll-like receptors (TLR), fundamental sensor molecules of the host innate immune system [167,168]. However, like curcumin, both in humans and in animal models, resveratrol has been associated with poor bioavailability (less than 1%) due to its poor aqueous solubility and its extensive metabolism in the intestine and liver [169,170]. After administration, this molecule undergoes rapid phase I and phase II metabolism to generate the main metabolites resveratrol-glucuronide and resveratrol-sulphate. These changes reduce cell permeability and consequently promote rapid excretion [171].

To overcome these limitations, NP-based formulations have been developed to enhance its bioavailability and to deliver the optimal resveratrol lung concentration. De Oliveira and colleagues demonstrated in a mouse model that oral administration of resveratrol via biodegradable lipid-cored polymeric nanocapsules optimizes its anti-inflammatory effect and prevents acute lung injury by allowing the molecules to be administered at lower doses than those administered in bulk form [165]. In particular, they demonstrated that nanoformulated resveratrol has protective effects at the lung level via reducing histological changes caused by hyper-inflammation and inflammatory leukocyte infiltration in bronchoalveolar fluid. Similarly, the study presented by Liu’s group showed that the use of polymeric NPs in a systemic inflammation mouse model allows the obtention of higher resveratrol bioavailability, facilitating intestinal absorption and increasing the half-life at the plasma level [172]. This is followed by the strong anti-inflammatory activity of inhibiting nitric oxide, IL-1β, IL-6, and TNF-α production, and interfering with the MAPK enzyme’s phosphorylation.

Moreover, because of its anti-inflammatory role and its vast therapeutic potential, resveratrol use has been implemented in COVID-19 treatment for patients with poor prognosis due to cardiovascular complications. In particular, in humans, resveratrol was shown to regulate the renin–angiotensin system and expression of angiotensin-converting enzyme-2 (ACE-2), which is involved in cardiovascular failure, making it a molecule with an excellent heart-protecting role [173,174,175]. For this reason, many formulations based on nanosystems, such as liposomes, lignin-based NPs and polymeric NPs, nanoemulsions, and inorganic NPs, have been widely tested in a variety of studies [174,176,177].

In addition to the demonstrated effect on the therapeutic potential of resveratrol, nanoformulations allow a reduction in some side effects associated with the oral administration of the polyphenol in native form. Resveratrol, indeed, has been shown to potently inhibit the gastrointestinal enzyme cycloxygenase-1 (COX-1), irritating the gastric mucosa. Animals that were administered resveratrol-loaded lipid-core nanocapsules showed significantly less damage than those administered free resveratrol, demonstrating its gastrointestinal safety [178].

### 5.3. Quercetin

The word “Quercetin” comes from the Latin term “*Quercetum*” which means “oak forest”, because it is firstly isolated from roots and barks of these trees. Quercetin is an important natural flavonoid that is widely present in the plant kingdom, and quercetin flavonols (mainly as quercetin glycosides) are found in a variety of foods, including apples, berries, grapes, onions, tea and tomatoes, as well as many seeds, nuts, flowers, and leaves [179]. This compound is involved in so many plant-developing processes, such as seed germination or photosynthesis, and its medicinal role makes it a highly regarded molecule [180]. Quercetin has been known for years for its anti-oxidative, anti-inflammatory, anti-proliferative, and immunoregulatory properties that prove its role as a valid compound to be included in nutraceuticals and food supplements [181].

Several studies and clinical trials, indeed, have highlighted that the quercetin-mediated anti-inflammatory mechanism is based on the inhibition of lipid peroxidation and arachidonic acid metabolism, modulating the release of pro-inflammatory mediators such as lipoxygenase, prostaglandins, leukotrienes, and cytokines [182,183,184,185]. The combination of these actions, but in particular the effect on the release of pro-inflammatory cytokines, have proved to be important factors in counteracting hyper-inflammation at the lung level induced via respiratory virus infection, making the flavonoid an interesting subject of several studies in the virology field [186]. Recently, it has been also shown that quercetin acts as a potent antiviral molecule by inhibiting the replication of several respiratory viruses, including Influenza virus, RSV, adenovirus and rhinovirus, in a dose-dependent manner. Although its antiviral mechanisms are still not well-understood, it is now clear that it simultaneously works at different stages of the viral life cycle. In vitro studies on Influenza A, H1N1 virus and rhinovirus confirmed that quercetin is both able to block viral endocytosis into the host cell and to inhibit viral RNA transcription by interfering with viral helicase and neuraminidase, finally stimulating viral clearance [187,188,189,190]. Given this high potential, during the last pandemic, several scientific groups around the world began to explore the effect of quercetin for treatment of COVID-19 also [191,192]. They proved that quercetin is able to bind Spike glycoprotein and inhibit its activity on the ACE-2 receptor, interrupting the virus cell’s host recognition and preventing SARS-CoV-2 infection [193]. Moreover, it can modulate the expression of several human genes targeted by the virus, of some proteases and of RNA-dependent RNA polymerase by inhibiting viral replication [194].

Despite the potential benefits, the low aqueous solubility, poor permeability, and instability in the physiological environment have limited quercetin’s widespread application in the pharmaceutical field [195]. Therefore, in recent years, several nanotechnology-based approaches have been developed to overcome these limitations. It has been widely demonstrated that quercetin encapsulation in liposomes improves its solubility in water, prolonging blood circulation times and increasing its bioavailability in lungs. As a result, the use of liposomal quercetin allows a reduction in the administered dose compared to that of native quercetin, decreasing the possibility of side effects in murine models [196]. In addition, the quercetin liposome was found to be able to decrease total cell counts and inflammatory cell proportions in bronchoalveolar lavage fluid, to reduce plasma TNF-α and transforming growth factor (TGF)-β1 concentrations and to increase superoxide dismutase (SOD) and glutathione peroxidase (GSH-PX) antioxidant agents in lung tissues of a murine model [197]. Pharmacokinetic benefits were also sought by preparing quercetin-loaded solid lipid systems as respiratory drug delivery vehicles. These systems allow the observation of an enhanced, slow, targeted release of the molecule owing to more efficient cellular internalization, also reducing the extent of degrading metabolism compared to that with free quercetin [198,199].

Moreover, a very interesting in vitro study was carried out by Neufurth et al. in 2021 on quecetin-encapsulated polymeric NPs for COVID-19 treatment [200]. These polymeric NPs are made by inorganic polyphosphate (polyP), a natural polymer which is secreted by platelets with the airway mucus in the respiratory system and represents a first-line defense against viral infections. This protective system is strongly impaired in COVID-19 patients. In vitro analysis in a cellular model of the pulmonary epithelium showed that these quercetin polymeric NPs were more effective than quercetin’s bulk form was in restoring mucus protein expression. It was shown to be particularly effective on the MUC5AC protein, a mucin that is highly present in the secreted gel-forming mucus and responsible for driving the mucus clearance system to remove virus/airborne particles from the lung. In this study, therefore, the use of a NP technology, in addition to the beneficial effect on quercetin pharmacokinetica, plays a fundamental role in improving its therapeutic properties in unbalanced mucin production and amplifies antiviral defense in this epithelium.

### 5.4. Vitamin C

L-ascorbic acid, better known as vitamin C, reduces oxidative damage in cells and improves several physiological activities in humans. Because of a gene mutation which encodes an enzyme (gulonolactone oxidase) involved in the biosynthesis of this vitamin, mammals are not able to produce it. The main source of vitamin C for humans is citrus foods, but they may also take its synthetic version through supplements. The normal plasma value of the vitamin should be around 50 µmol/L; this amount is able to prevent scurvy but would not be enough during a viral infection or under physiological stress [201]. This vitamin is well-known for its antioxidant properties but in recent years its antimicrobial, antiaging and immunomodulatory effects have also been discovered [202].

It has been known for years that patients with severe conditions, such as sepsis or multiple organ failure, have extremely low levels of vitamin C. This correlation has also emerged in patients with severe symptoms of SARS-CoV-2 infection, who are hospitalized in intensive care unit with respiratory distress [203]. For this reason, several clinical studies have explored the effects of vitamin C supplementation in the acute stages of pathology management. They demonstrated that high-dose intravenous administration of this vitamin is responsible for reducing serum markers of inflammation and lung damage, counteracting the development of ARDS and decreasing the mortality and length of stay of infected patients [204]. In vitro studies on different inflammatory environments have shown that vitamin C acts against pro-inflammatory cytokines in two different ways. In its reduced form as an ascorbate, it can directly scavenge ROS inhibiting ROS-mediated NF-κB signaling [205,206], while in its oxidized form as a dehydroascorbate produced following the ROS scavenging mechanism, it inhibits the kinase activity involved in the NF-κB pathway, such as that of IκBα and β, and in the MAPK cascade, such as that of P38 kinase [207,208,209,210]. However, it has been shown that the effect of vitamin C as an anti-inflammatory molecule is dependent on the cell type and organ involved, interfering with different molecular pathways in various health conditions. Some recent studies, indeed, have suggested that in respiratory virus-induced sepsis and pneumonia, the molecule interacts with several mediators including the epidermal growth factor (EGF), c-jun proto-oncogene, C-C chemokine receptor type 5 (CCR5), angiotensin II receptor type-2 (AGTR-2), and signal transducer and transcription activator 3 (STAT3) [201,211,212]. In addition, these interesting effects are correlated with effective antiviral activity, making this vitamin a good remedy against respiratory viral infections. Vitamin C has been reported to have protective effects against a number of respiratory viruses, such as SARS-CoV-2, IAV H1N1 and H3N2, RSV and other viruses responsible for common respiratory illnesses [213]. Again, it acts with multiple mechanisms; it is capable of damaging the viral capsid thanks its redox properties and of inhibiting viral replication by creating an unfavorable environment for this activity and by directly interfering with replication enzymes. In addition, vitamin C causes the degradation of single- and double-stranded genomes of RNA and DNA viruses, resulting in reduced production of viral proteins [214].

It is important to note that vitamin C absorption, distribution, and metabolism in humans are all very complex processes. Most of the intestinal absorption and distribution in the body is regulated by the sodium-dependent vitamin C protein family (SVCT) that co-transports sodium and ascorbate ions across membranes, and their presence is fundamental because the hydrophilic nature of vitamin C limits its passive diffusion through biological membranes. However, SVCT expression and the affinity for vitamin C varies between the organs and so the vitamin C pharmacokinetics are non-linear in the body. Moreover, while low-molecular-weight molecules are typically metabolized by phase I and II enzymes, vitamin C is involved in numerous physiological redox reactions. The ascorbate is oxidized to the ascorbil radical, which subsequently undergoes a dismutation reaction to re-form the ascorbate and generate dehydroascorbic acid. This is then reduced to ascorbate through enzyme reactions, which are highly efficient in healthy individuals, while in the presence of pathologies, this process is altered, making it necessary to increase the administered dose of vitamin C. Consequently, high plasma levels of the vitamin can be achieved only via intravenous injection and not via oral administration, representing a strong limit and its impracticability for patients [215,216,217]. For these reasons, several vitamin C delivery nanocarriers have been proposed to enhance the delivery performance of vitamin C compared to that of its native form [202]. A clinical study by Davis et al. showed that vitamin C encapsulation in liposomes, when orally administered to patients with a pathological condition characterized by high oxidative stress, significantly increases the molecule’s blood bioavailability, even if without greater clinical efficacy compared to that in the unencapsulated form [218]. This advantage has also been confirmed in a study by Prantl and co-workers in which vitamin C-containing liposomes were monitored in real time via ultrasound in the gastrointestinal tract of four healthy volunteers [219]. They observed a high uptake trough the duodenum and jejunum of liposomes, its good adherence to the intestinal wall, penetrating through the mucosa, and then its ability to reach the mesenteric vessels and liver in higher concentrations [219]. Indeed, clinical trials showed that oral liposomal vitamin C may be better at reducing the extent of multiple organ failure and may better improve the short-term outcomes of sepsis, counteracting the cytokine storm by inhibiting the production and release of pro-inflammatory cytokines IL-1β, IL-2, IL-6, and TNF-α from human monocytes of COVID-19 patients with severe symptoms [220]. Similarly, the antioxidant, anti-inflammatory and antiapoptotic activity of vitamin C NP formulation was demonstrated also in a mouse model with renal failure [221]. This can be an additional therapeutic approach against multiple organ failure resulting from ARDS following respiratory viral infections. Indeed, some people suffering for severe symptoms of COVID-19 show signs of renal impairment that often result in an unfortunate outcome of the infection [222]. In this research, vitamin C NPs were shown to significantly reduce some oxidative stress parameters such as malondialdehyde (MDA), SOD and catalase (CAT) and blood inflammatory parameters including NF-κB, IL-1β and TNF-α. In addition, they were also able to modulate, more than the bulk form was able to, the renal expression of Nrf2, Bcl-2, caspase-3, BAX and mTORc1, which are apoptosis markers responsible for renal damage.

## 6. Conclusions

Natural substances have been used for decades for their therapeutic properties against a number of different pathologies. Consequently, these substances have been “reproposed” in recent years in several in vitro and in vivo studies as a resource with which to counteract hyper-inflammation and the cytokine storm induced by several viral respiratory infections, which represent one of the most common causes of death worldwide [15]. However, poor water solubility and low bioavailability are some of the drawbacks of natural products, limiting their widespread clinical application. In this context, nanotechnology has made it possible to bypass these specific limitations, ensuring that the therapeutic effects of molecules remain unaltered, increasing cellular uptake, and thus requiring lower administration doses.

**Figure 4:**
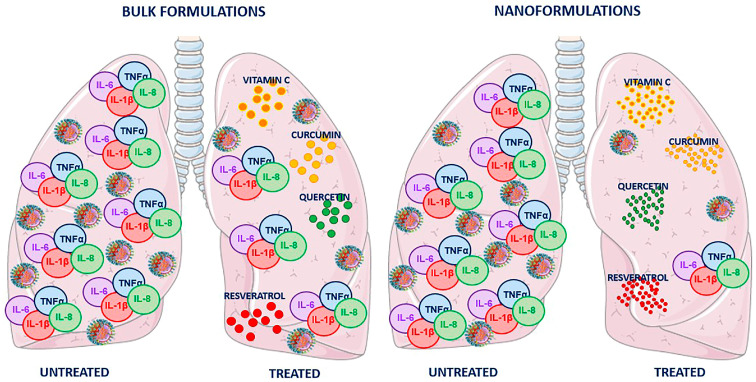
Schematic representation of lung virus-induced cytokine storm and the effects of curcumin, resveratrol, quercetin, and vitamin C in bulk or nanoform. Two pairs of lungs infected with respiratory virus, responsible for the induction of cytokine storm with a severe increase in IL-6, IL-8, TNF-α and IL-1β proinflammatory cytokines, are shown. In both pairs, the left lung represents the condition in the absence of treatment, while in the right lung, the effectiveness of treatment with natural compounds is shown. The nanoformulations result in more successful counteraction of the cytokine storm (reducing IL-6, IL-8, TNF-α and IL-1β pulmonary levels) and viral infection, compared to the bulk formulations.

Moreover, nanotechnological approaches, polymer NPs, liposomes, micelles, and solid lipid NPs are feasible, inexpensive, biocompatible, represent a suitable strategy for managing respiratory viral infections especially, and are increasingly analyzed in different studies as an innovative resource for lung-targeted drug delivery. Here, the main results published in recent years on curcumin, resveratrol, quercetin, and vitamin C have been examined. They provide promising scientific evidence of the nanoformulation benefits in increasing the natural substance’s therapeutic potential to prevent and/or control the occurrence of severe clinical complications associated with respiratory viruses, such as the lung cytokine storm (Figure 4). Up to date, some studies have already been published in the literature that describe the benefits of using nanosystems in formulations containing natural substances, due to their higher bioavailability, which leads to lower administered doses, and less side effects; in other words, their improved pharmacokinetic properties. Although the preliminary results obtained with the combination of repositioned natural compound and nanoformulations are very promising, this represents a hot topic and a rapidly growing field in drug delivery, and much of the research is still ongoing. Considering that in the literature. mainly in vitro and in animal model studies are present, more clinical trials on humans are needed, allowing a significant transition from “bench to bed”.

## Figures and Tables

**Table 1:** Inflammatory response to respiratory viral infections.

Virus	Inflammatory Response	Reference
IAV	Elevated levels of pro-inflammatory cytokines are associated with poor prognosis. Infection with A/H5N1 virus or the pandemic H1N1 2009 virus induces higher levels of IL-6, TNF-α, IFN-γ and chemokines in patient sera, particularly in fatal cases, compared to seasonal strains of IAV.	[36,37,38]
SARS-CoV-2	Secretion of pro-inflammatory cytokines and chemokines (including IL-6, Interferon gamma inducible protein-10, macrophage inflammatory protein 1α and β, and monocyte chemoattractant protein-1) that attract monocytes, macrophages, and T-cells to the site of infection, promote further inflammation (with the addition of T-cell-produced IFN-γ) and establish a pro-inflammatory feedback loop. The cytokine storm circulates to other organs, causing multi-organ damage. Interferon gamma-inducible protein-10 and monocyte-chemoattractant protein-1 are biomarkers associated with the severity of COVID-19 disease.	[39,40,41]
RSV	IL-8-mediated cellular response leads to lung inflammation and tissue damage. Humoral response is characterized by the production of several Th1 and Th2 cytokines and chemokines. Elevated levels of Th2 cytokines, particularly IL-6, are associated with patients with bronchiolitis or pneumonia requiring hospitalization.	[42,43,44,45]
Rhinovirus	Secretion of pro-inflammatory cytokines, such as IL-6 and IFN-γ, and chemokines such as C-C motif ligand 5 and IL-8, which drive the recruitment of immune cells to the site of infection, contributing to tissue damage.	[46,47]

**Table 2:** Organic NP system description.

Type	Description	Pharmacokinetic Improvements	References
Polymeric NPs	Colloidal particles of natural or synthetic polymer matrices constructed with various designs/sizes. Size: 10–100 nm	Prolonged bloodstream circulation and systemic exposure. Protection from drug degradation. Enhanced cellular permeability. Different administration routes. Delayed drug clearance.	[105]
Liposomes	Self-assembled phospholipid vesicles of concentric lipid bilayers that enclose a central aqueous compartment. Size: 50–100 nm	Prolonged bloodstream circulation and systemic exposure. High drug solubilizing potency. Protection from drug degradation. Enhanced cellular permeability. Delayed drug clearance.	[106]
Micelles	Autonomous aggregated colloidal structures with a hydrophobic core and hydrophilic shell. Size: 10–100 nm	Prolonged bloodstream circulation and systemic exposure. High drug solubilizing potency. Protection from drug degradation. Enhanced cellular permeability. Delayed drug clearance.	[107]
Lipid NPs (solid lipid NPs and nanostructured liquid carriers)	Lipophilic particles with a lipid core matrix stabilized by surfactants or emulsifiers. The lipid matrix is made by solid lipids in solid lipid NPs or solid lipids and liquid oils in nanostructured liquid carriers. Size: 100–400 nm	Prolonged bloodstream circulation and systemic exposure. High drug solubilizing potency. Protection from drug degradation. Enhanced cellular permeability. Specific drug delivery. Different administration routes.	[108]

## Data Availability

Not applicable.
